# Citizen science data as a tool for monitoring moss diversity in Poland: potential and limitations

**DOI:** 10.1007/s10661-026-16092-5

**Published:** 2026-10-26

**Authors:** Mikołaj Latoszewski, Paweł Świsłowski, Małgorzata Rajfur, Grzegorz J. Wolski

**Affiliations:** 1 Faculty of Biology and Environmental Protection, Department of Geobotany and Plant Ecology, University of Lodz, ul. Banacha 12/16, Lodz, Poland; 2 Institute of Biology, University of Opole, ul. Kominka 6/6a, Opole, 45-032 Poland

**Keywords:** Citizen science, iINaturalist, Biodiversity monitoring, Gini coefficient, Sampling bias

## Abstract

In this study, the potential and limitations of iNaturalist for monitoring mosses in Poland (2019–2025) were assessed using 17,451 verified “Research Grade” records. Rarefaction enabled diversity pattern analysis while accounting for sampling effort differences. Observational activity peaked in early-spring (March) and autumn (October). Analysis revealed extreme asymmetry in research effort (*G*_users_ = 0.89): the 12 most active users contributed 65.5% of national records, creating a “super-user effect.” Rarefaction corrected this bias, identifying the Pomeranian Voivodeship as having the highest standardized diversity (*S*_rar_ = 80.9). Citizen science effectively supports specific moss monitoring aspects. Observers documented 151 unique records of the alien moss *Campylopus introflexus*, representing over 60% of sites recorded during 30 years of traditional floristic research. Despite the “microscope barrier” restricting the database largely to visually identifiable bryophytes (e.g., reducing nearly the entire genus *Hypnum* to *H*. *cupressiforme*; *G*_taxa_ = 0.76), iNaturalist serves as a crucial early-warning system. Ultimately, large-scale citizen scientists datasets provide valuable guidance for identifying priority areas for future bryological research.

**Supplementary Information:**

The online version contains supplementary material available at https://doi.org/10.1007/s10661-026-16092-5.

## Introduction

Mosses constitute one of the oldest and most important groups of land plants, playing key roles in the functioning of forest and peatland ecosystems (Vanderpoorten & Goffinet, 2009). They contribute significantly to water retention and to the biogeochemical cycles of carbon and nitrogen, while also providing microhabitats for numerous invertebrate fauna (Gerson, 1982; Turetsky, 2003) and fungi (Davey & Currah, 2006). Due to their poikilohydric nature and sensitivity to environmental changes, mosses are also regarded as excellent bioindicators (Andersen et al., 1978; Rajfur et al., 2026; Świsłowski et al., 2026; Wolski et al., 2026).

Despite their ecological importance, mosses remain an insufficiently studied group compared to vascular plants (Deilmann et al., 2024; Vanderpoorten & Goffinet, 2009; Wolski & Kruk, 2020). Traditional bryological monitoring is a time-consuming and costly process that requires advanced taxonomic expertise and fieldwork conducted by a limited number of specialists. This phenomenon, referred to in the literature as the taxonomic impediment, results in an incomplete knowledge of the current distribution of many species (Wheeler et al., 2004).

In recent decades, the dynamic development of citizen science has emerged as a response to these limitations. Open-access biodiversity platforms such as iNaturalist enable the large-scale collection of biodiversity data by citizen scientists and nature enthusiasts (Theobald et al., 2015). The development of mobile applications, widespread Internet access, and, above all, the ability to rapidly produce high-quality digital photographs have created a unique space in which citizens can become active contributors to scientific knowledge rather than merely passive recipients of it (Hognogi et al., 2023). Owing to advances in digital photography and artificial intelligence algorithms supporting species identification, the number of occurrence records of various organisms in digital databases has increased rapidly (Wäldchen & Mäder, 2018). However, this also raises questions regarding data quality and spatial representativeness, particularly in the context of so-called sampling bias (Geldmann et al., 2016; Isaac & Pocock, 2015; Kosmala et al., 2016).

The specificity of bryophyte taxonomy—including mosses—constitutes one of the greatest challenges within botanical citizen science projects. While many moss species can be readily identified in the field using macroscopic characters or a hand lens, definitive species-level identification within taxonomically challenging groups relies heavily on microscopic characteristics (Glime, 2017). This presents a distinct contrast to most vascular plants, whose identification is typically based on macroscopic generative organs (flowers and fruits). Consequently, many modern identification keys rely on features invisible to the naked eye (Smith, 2004).

Among the critical features determining accurate identification within many genera (e.g., *Bryum*, *Plagiothecium*, and *Orthotrichum*) are the shape and dimensions of cells, the degree of leaf margin serration, the presence and structure of the costae, and, above all, the dimensions of leaf cells (Holyoak, 2004; Wolski, 2025). In many taxonomic groups, definitive identification requires precise cross-sections of the leaf or stem to be prepared and examined (Ignatova et al., 2025; Zander, 1993).

This aspect creates significant limitations for platforms such as iNaturalist, which rely almost exclusively on the general macroscopic appearance of plants. The inability to verify microscopic diagnostic features means that most citizen scientists observations are restricted to the so-called “visual fraction”—taxa with large gametophytes and distinctive macroscopic traits. This situation is further exacerbated by the phenomenon of plant blindness, which in the case of bryophytes takes the form of extreme perceptual reduction, whereby the diverse bryoflora is perceived as a visually homogeneous group lacking distinguishing features (Cerrejón et al., 2024; Wandersee & Schussler, 1999). Consequently, without the support of specialized optical equipment and taxonomic expertise, citizen science data may lead to a systematic underestimation of the true biological diversity within taxonomically critical groups.

The aim of this study is to analyze spatial patterns of moss diversity in Poland between 2019 and 2025 using verified (“Research Grade”) data from the iNaturalist platform. We addressed four simplified research questions corresponding to the structural data divisions: (1) What is the taxonomic and seasonal structure of iNaturalist moss data in Poland? (2) How do spatial sampling bias and the “super-user effect” distort regional alpha and beta diversity estimates? (3) To what extent can opportunistic citizen science data detect rare, indicator, and invasive species? (4) What is the reliability of photographic identification given the constraints of the "microscope barrier"? This study evaluates the potential of digital biodiversity databases to complement traditional bryological knowledge in Poland.

## Materials and methods

### Data source and selection

In this study, the term “mosses” strictly refers to true mosses belonging to the phylum Bryophyta. Other bryophyte groups, namely, liverworts (Marchantiophyta) and hornworts (Anthocerotophyta), were completely excluded. The analysis includes the class Sphagnopsida (*Sphagnum*), treated here as a distinct structural component of the visual fraction of data.

The material basis of this study consisted of moss occurrence records from Poland obtained from the iNaturalist platform (iNaturalist, 2026). The raw dataset was downloaded and exported directly within the R computational environment and included observations recorded from 1 January 2019 to 31 December 2025. The complete raw dataset retrieved from the platform is provided in Supplementary Material S1. To ensure data reliability, the raw dataset was subjected to filtering procedures. Only observations with “Research Grade” status were included. On the iNaturalist platform, this data quality designation is automatically awarded to observations that contain a media file (photo or sound), a valid date, and precise geographic coordinates, while also achieving a community consensus where more than two-thirds of the identifiers agree on the taxonomic identity. This approach represents a common and robust method for reducing verification errors in citizen science projects (Kosmala et al., 2016).

To assess the reliability of species identifications, interviews were conducted with the 12 most active observers, who were responsible for 65.5% of all Research Grade records nationwide. Communication with respondents and the distribution of the structured questionnaire were carried out through the built-in private messaging system on the iNaturalist platform. The survey was based on a structured questionnaire concerning (1) the use of optical equipment (microscope/hand lens), (2) the use of bryological literature, (3) fieldwork strategies, and (4) formal educational background. This approach made it possible to characterize the documentation practices employed by the most active moss observers in Poland.

In addition, a manual verification was conducted on a random sample comprising 9.4% of the dataset (*n* = 1654). The sample size was not arbitrarily defined as exactly 10%, but resulted from the adoption of a fixed number of records selected for validation and its subsequent relation to the total number of observations in the dataset. Consequently, the final proportion of the sample should be regarded as approximate and reflective of the actual data structure rather than a predetermined percentage-based sampling scheme. Each specimen included in the sample was re-identified on the basis of photographic documentation, taking into account all diagnostic features visible in the images. The results of this verification were used to calculate the overall taxonomic error rate for the entire dataset.

Spatial data were restricted to the administrative boundaries of Poland (GADM, 2026). All spatial records were originally stored in the WGS84 coordinate system (EPSG:4326), and spatial analyses were conducted using the sf package after transformation into coordinate systems compatible with the administrative data. Scientific names were standardized to binomial species-level names by retaining only complete species-level identifications extracted from iNaturalist records. Records identified only to higher taxonomic ranks (e.g., genus level) or containing incomplete species names or variates were excluded from further analyses. This approach ensured consistency of taxonomic resolution across the dataset and reduced fragmentation of the species matrix in subsequent analyses. This decision was motivated by the limited sample size and the highly uneven distribution of records at taxonomic levels below species, which would have led to excessive fragmentation of the data matrix, an increased proportion of singleton observations, and instability in diversity estimators and multivariate analyses (NMDS). The adopted approach allowed for more stable and comparable estimates of community structure at the regional scale.

To reduce pseudoreplication bias, duplicate records were removed, defined as multiple occurrences of the same taxon at the same location. The raw dataset retrieved for Poland was systematically reduced using a rigorous quality control workflow to eliminate incomplete identifications, coordinate errors, and pseudoreplication (Table 1). Species represented by a single observation (singletons; 45 species) were individually reviewed for taxonomic plausibility (the detailed list of these records is provided in Supplementary Table S2). While we recognize that singletons can represent genuinely rare taxa of high biological importance for biodiversity monitoring, they were excluded from the primary analytical matrix. This exclusion was a strictly statistical procedure aimed at preventing severe noise and instability in diversity estimators and multivariate ordination (NMDS), which are highly sensitive to extremely rare occurrences. Because this filtering affected less than 0.3% of all observations, it ensured robust and stable statistical comparisons across regions. However, to preserve their monitoring value, all singletons are fully documented in the supplementary materials, and notable credible records (e.g., *Splachnum ampullaceum*) are addressed in the discussion. The final dataset used for spatial and statistical analyses comprised 17,451 records.

**Table 1:** Stepwise data filtering and quality control workflow for the iNaturalist moss dataset in Poland (2019–2025)

Filter step	Methodological procedure	Remaining records (*N*)
Step 1: raw data	Total mosses occurrences downloaded for Poland	37,223
Step 2: quality grade	Restriction to verified observations with “Research Grade” status	18,073
Step 3: temporal filter	Limitation to the target calendar years (1 January 2019–31 December 2025)	17,738
Step 4: taxonomic clean	Removal of incomplete, genus-level identifications (e.g., *Bryum* sp.) and missing coordinates	17,727
Step 5: spatiotemporal dedup	Removal of exact duplicate records (same species, location, and date) to reduce pseudoreplication	17,528
Step 6: singleton removal	Manual review and exclusion of species represented by a single observation	17,483
Step 7: spatial clipping	Spatial intersection with GADM boundaries and exclusion of out-of-bounds coordinate errors	17,451

### Spatial and statistical analyses

All analyses were performed in R v. 4.4.2 (R Core Team, 2024), using the following packages: tidyverse (Wickham et al., 2019), sf (Pebesma & Bivand, 2023), vegan (Oksanen et al., 2024), ineq (Zeileis & Kleiber 2014), and viridis (Garnier, 2024). The distribution of sampling effort was visualized using hexagonal binning on a logarithmic scale (Wickham, 2016). The complete R script used for raw data cleaning, analyses, and visualization is available in Supplementary Code S1.

Standardized species richness (*S*_rar_) was estimated using individual-based rarefaction with a fixed sample size of *n* = 300 observations per region. This value was chosen to optimize the trade-off between estimation stability and geographic coverage of the analyses. The rarefaction threshold of *n* = 300 observations per region was determined through an optimization approach aimed at maximizing the subsampling depth while preserving the highest possible number of administrative units (14 out of 16 voivodeships). Setting a higher threshold would result in the immediate exclusion of additional regions due to insufficient sample sizes, whereas a lower value would unnecessarily reduce the statistical power and data saturation of the diversity estimates for the qualifying units. Consequently, regions failing to meet this criterion were excluded from subsequent comparative analyses (Oksanen et al., 2024). Differences in species composition between regions were analyzed using non-metric multidimensional scaling (NMDS) based on a species abundance matrix. Ordination was performed using the metaMDS function from the vegan package, employing Bray–Curtis dissimilarity as the default metric of ecological community dissimilarity.

Bias in user activity (the so-called “super-user effect”) was quantified using Lorenz curves, the Gini coefficient (*G*) calculated for the distribution of the number of observations per user (*G*_users_), and the percentage contribution of the top regional observer (top observer). Similarly, unevenness in taxon frequency distribution was assessed using the Gini coefficient (*G*_taxa_) calculated for the number of observations per taxon (Boakes et al., 2016). Sampling completeness was additionally evaluated using species accumulation curves (SACs), generated through random permutations (100 iterations) using the specaccum function from the vegan package. The sampling unit consisted of aggregated spatial records defined by rounded geographic coordinates. Local diversity was also described using the Shannon index (*H*′), calculated for each administrative region based on species abundances.

## Results

### Data structure, seasonal dynamics, and observer community

**Fig. 1:**
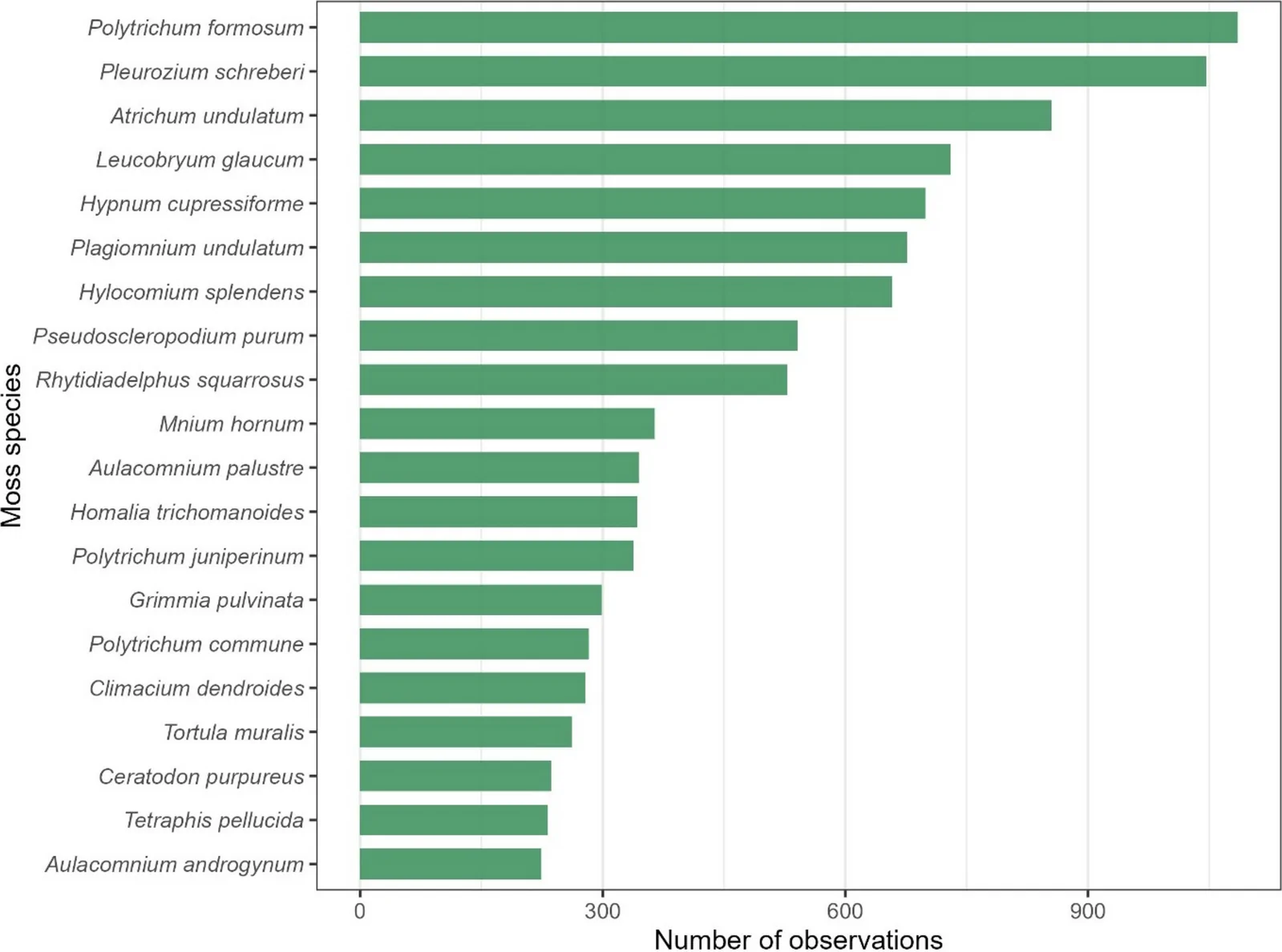
Ranking of the 20 most frequently recorded moss species in Poland based on iNaturalist data (2019–2025)

Analysis of the verified dataset (*n* = 17,451) revealed uneven taxon observation frequencies. The 20 most frequently recorded species accounted for 57.26% of all records. The most commonly documented taxa included *Polytrichum formosum* (*n* = 1085), *Pleurozium schreberi* (*n* = 1048), *Atrichum undulatum* (*n* = 856), *Leucobryum glaucum* (*n* = 731), and *Hypnum cupressiforme* (*n* = 699). Although the ranking is dominated by widespread forest species or taxa with a broad ecological amplitude (e.g., *H*. *cupressiforme*), the dataset also reflects intensive sampling of urban environments. This is evidenced by the high position of synanthropic taxa, e.g., *Tortula muralis*, recorded at 262 locations (Fig. 1).

**Fig. 2:**
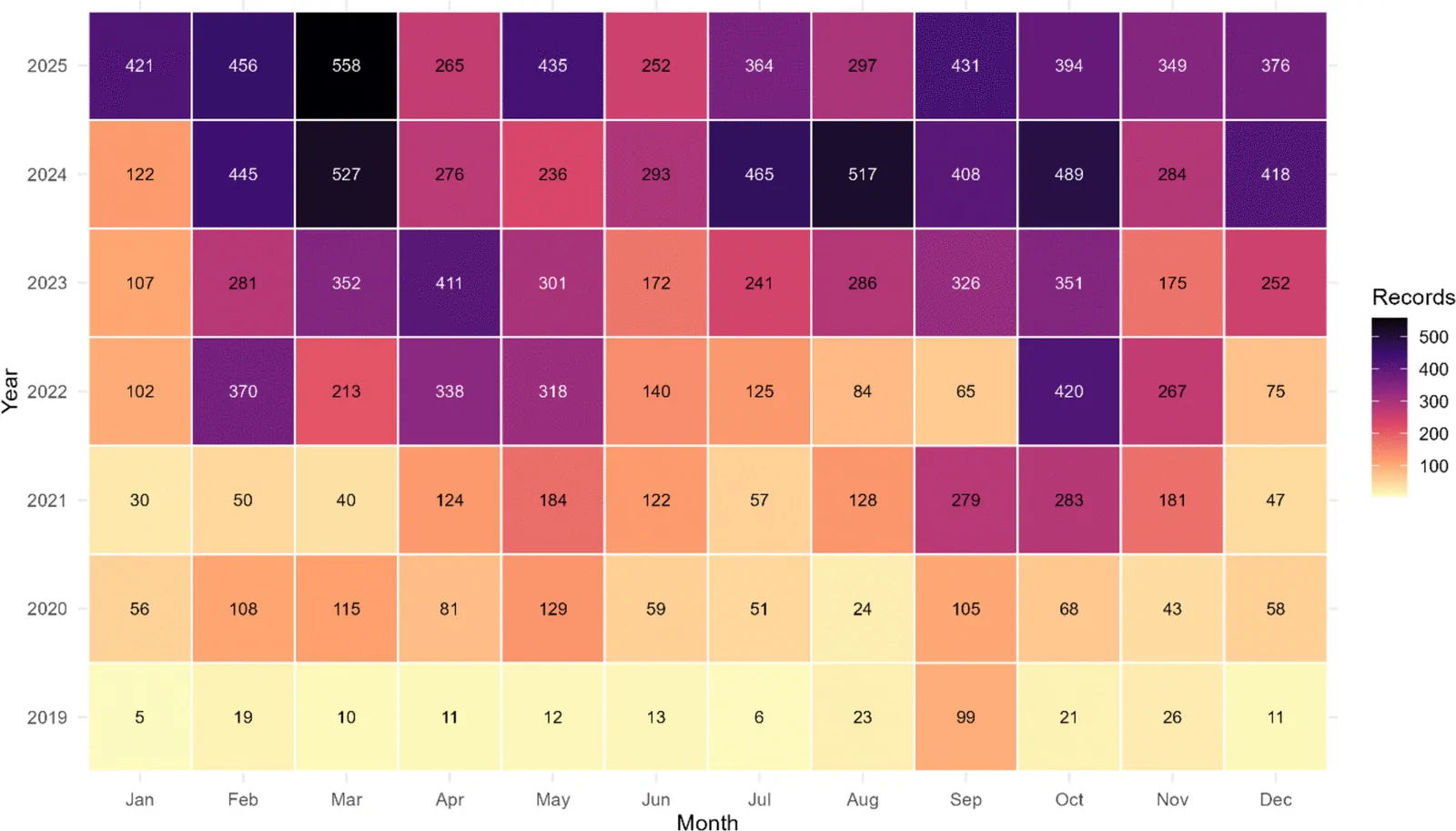
Seasonality and growth dynamics of the moss dataset on the iNaturalist platform (2019–2025)

The seasonal distribution (Fig. 2) shows two peaks of user activity per year. The highest number of records is observed in March, while a secondary peak occurs in October. In the final year of the study (2025), a noticeable increase in records was also observed during the winter months (January–March).

The dataset under analysis comprised 1117 observers. The distribution of their activity was characterized by a Gini coefficient of *G*_users_ = 0.89, indicating a highly unequal contribution structure. The top 10% most active users (*n* = 112) generated 87.1% of all Research Grade records, while 12 leading contributors alone accounted for 65.5% of the national dataset. The most active observer submitted over 3500 records, corresponding to approximately 20.2% of the entire database.

**Fig. 3:**
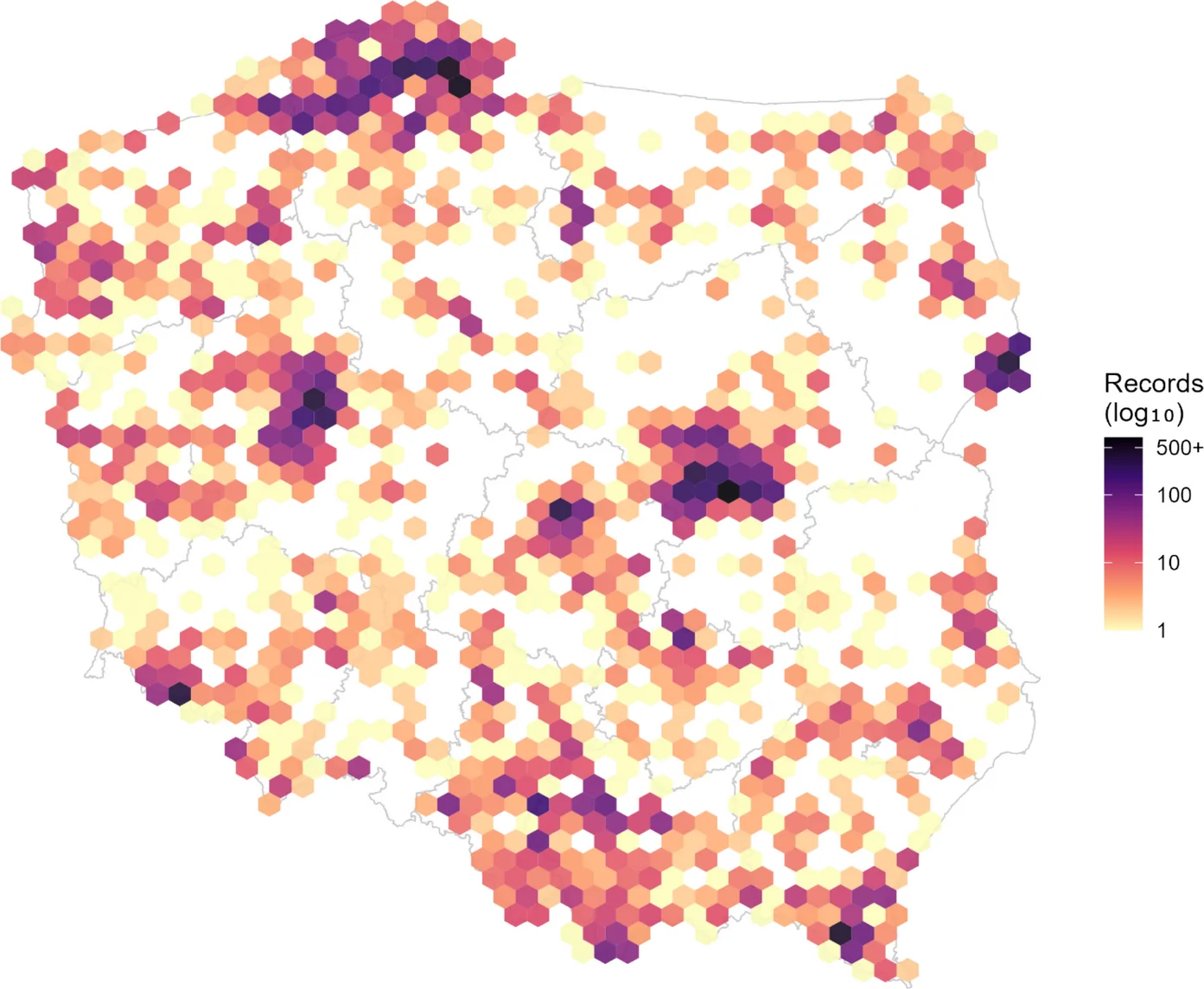
Spatial sampling effort of moss records in Poland based on iNaturalist data (2019–2025). Hexagonal aggregation of observation density (log scale)

**Fig. 4:**
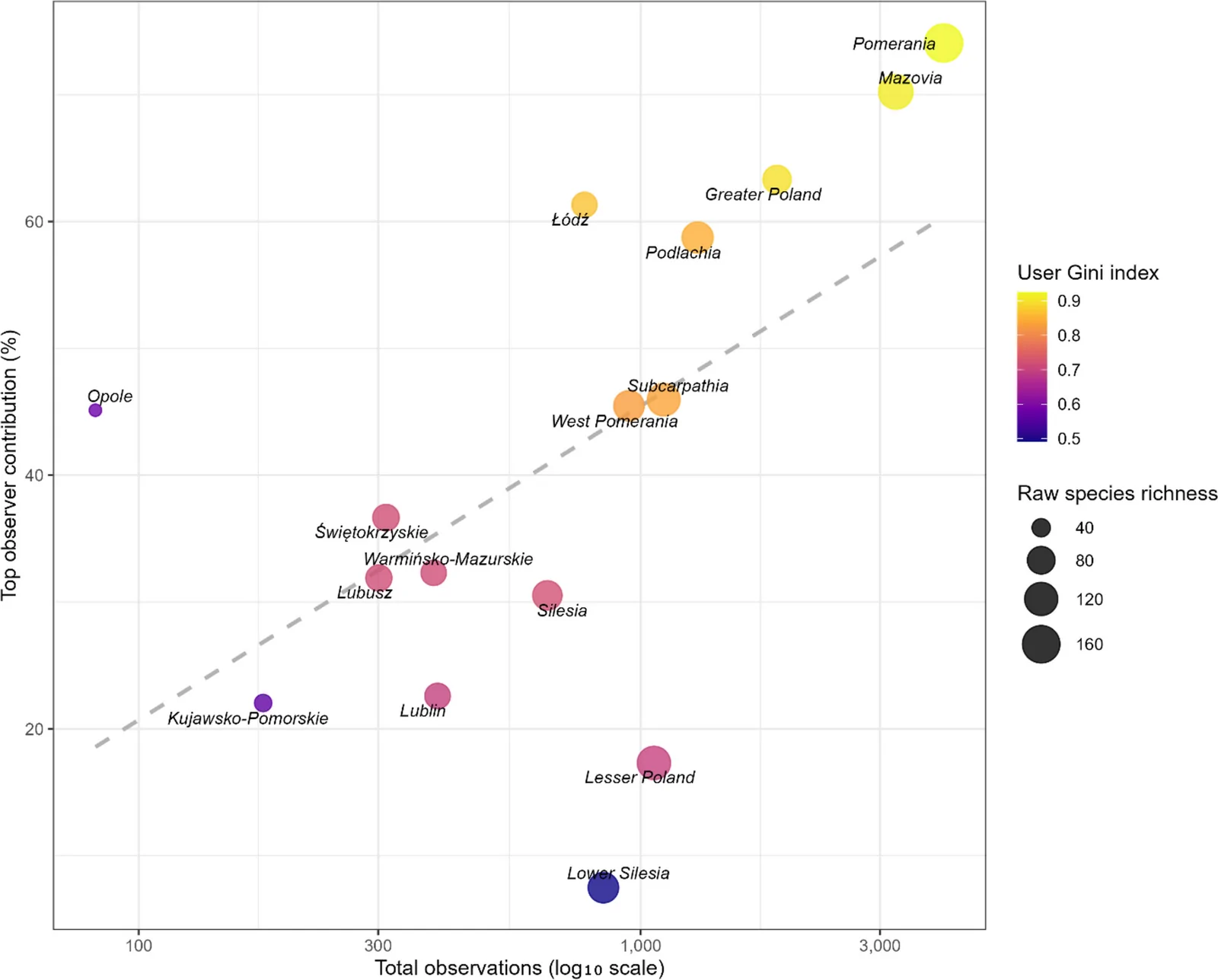
Relationship between the total number of observations and the percentage contribution of the top observer across individual voivodeships. Point size reflects raw species richness, while the color scale represents the Gini index for user activity within the region

The spatial distribution of observational effort was visualized using a hexagonal grid map (Fig. 3), which revealed a pronounced spatial heterogeneity in data coverage across the country. The mean share of the regional lead observer in the generation of voivodeship-level data was 41.59%. This value exhibited substantial territorial variation: the highest contributions of lead observers were recorded in the Pomeranian (74.1%), Masovian (70.2%), and Greater Poland (63.3%) voivodeships, whereas the lowest values were observed in Lower Silesian (7.5%), Lesser Poland (17.3%), and Kuyavian–Pomeranian (22%) voivodeships. This regional variation, along with its relationship to overall sampling effort, is further illustrated in Fig. 4.

**Fig. 5:**
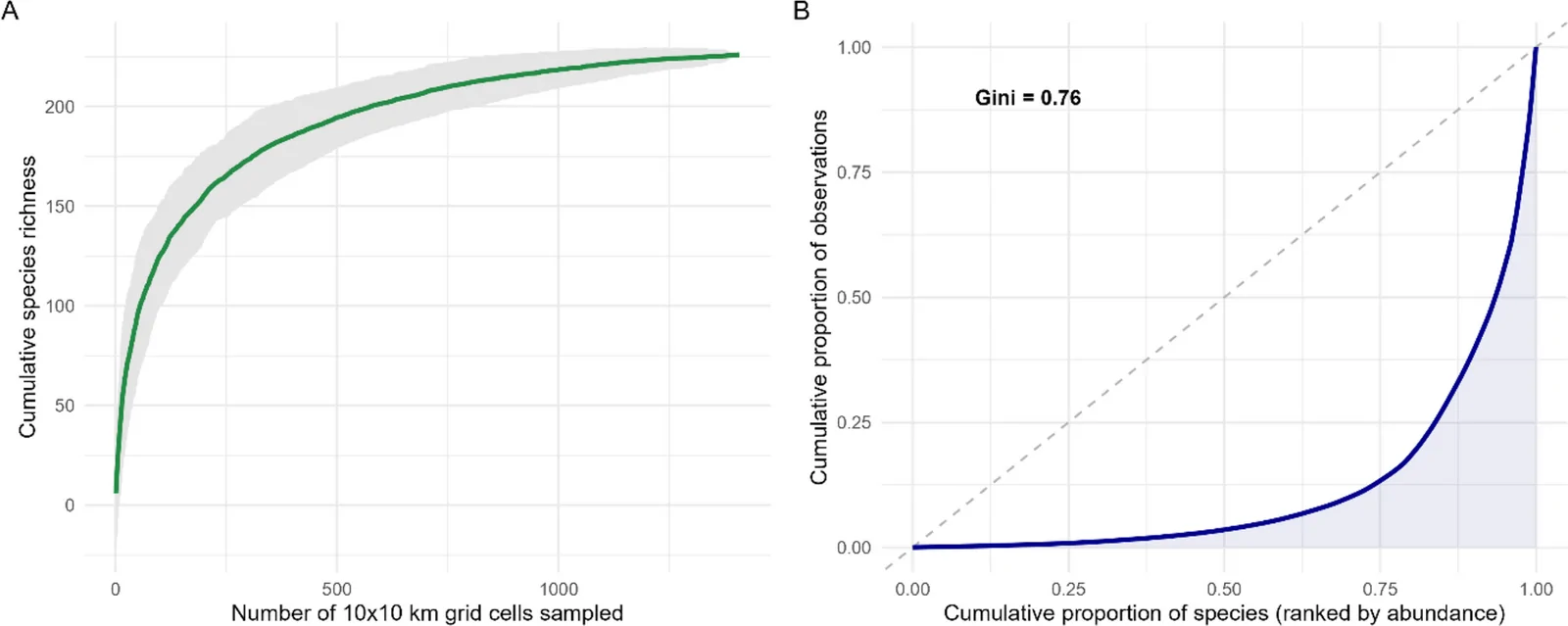
Assessment of data completeness and structure. **A** Species accumulation curve (SAC) generated using rarefaction. **B** Lorenz curve illustrates the inequality in taxon representation within the dataset (*G*_taxa_ = 0.76)

The species accumulation curve (SAC), generated using random permutations for the final sample of 17,451 records, exhibits an asymptotic trend. The Gini coefficient for the distribution of observations among taxa was *G*_taxa_ = 0.76, with a significant portion of the dataset comprising common species and taxa represented by singletons in the original matrix (45 species) (Fig. 5).

### Spatial distribution of biodiversity (alpha and beta diversity)

**Fig. 6:**
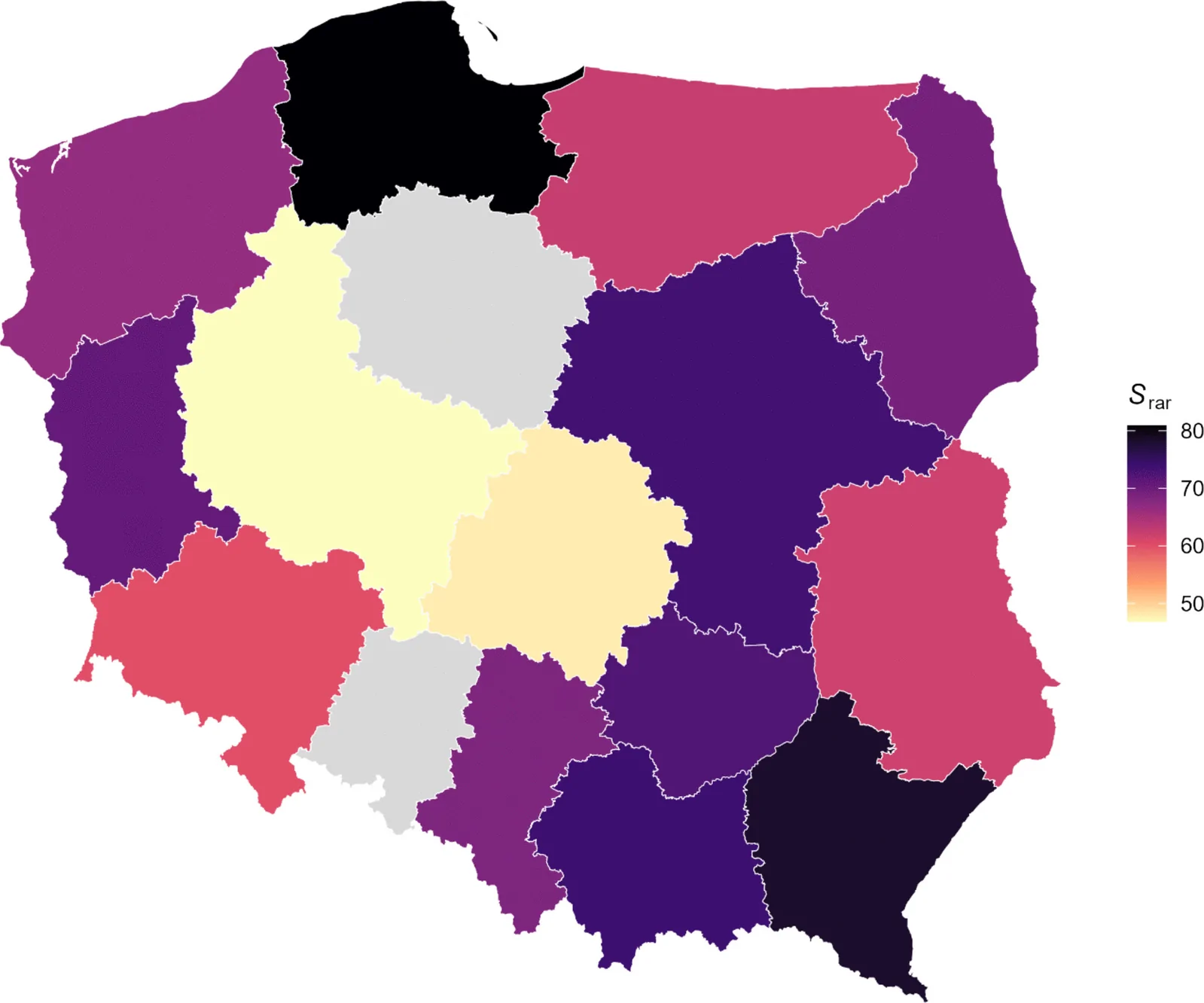
Standardized species diversity of mosses in Poland based on iNaturalist data (2019–2025). Species richness was calculated using rarefaction for a constant sample size of *n* = 300 observations per region. Gray areas indicate regions with insufficient data (*n* < 300)

Based on raw, unstandardized data, the highest species richness among Poland’s top-level administrative provinces (voivodeships) was recorded in the Pomeranian voivodeship (*S* = 168, *n* = 4015), where the Shannon index reached *H*′ = 4.22 (Supplementary Table S1). Spearman’s rank correlation analyses revealed a significant positive relationship between the total number of records (*N*) and the observed number of species (*S*) at the voivodeship level (*r*_s_ = 0.86, *p*<0.001). Fourteen voivodeships were qualified for the standardized diversity analysis and nonmetric multidimensional scaling (NMDS). The selection of the *N* = 300 Research Grade records threshold was dictated by optimizing the trade-off between estimation stability and geographical coverage. Adopting a higher threshold (e.g., *N* = 400) would have resulted in the exclusion of another region from the analysis. Due to failing to meet this requirement, the Kuyavian–Pomeranian (*N* = 177) and Opole (*N* = 82) voivodeships were excluded from comparative analyses (Fig. 6).

Following standardization via rarefaction (e.g., a constant sample size of *n* = 300), the highest species richness was again found in the Pomeranian voivodeship (S_rar_ = 80.9). The Subcarpathian voivodeship ranked second (*S*_rar_ = 78.3, *H′* = 4.21). In the Masovian voivodeship, despite a large volume of raw data (*n* = 3224), the standardized species richness was *S*_rar_ = 73.5. The lowest diversity parameters were recorded in the Greater Poland voivodeship (*S*_rar_ = 46.8, *H*′ = 3.31). Voivodeships that did not reach the *n* = 300 threshold (e.g., Opole) were excluded from standardized comparisons.

**Fig. 7:**
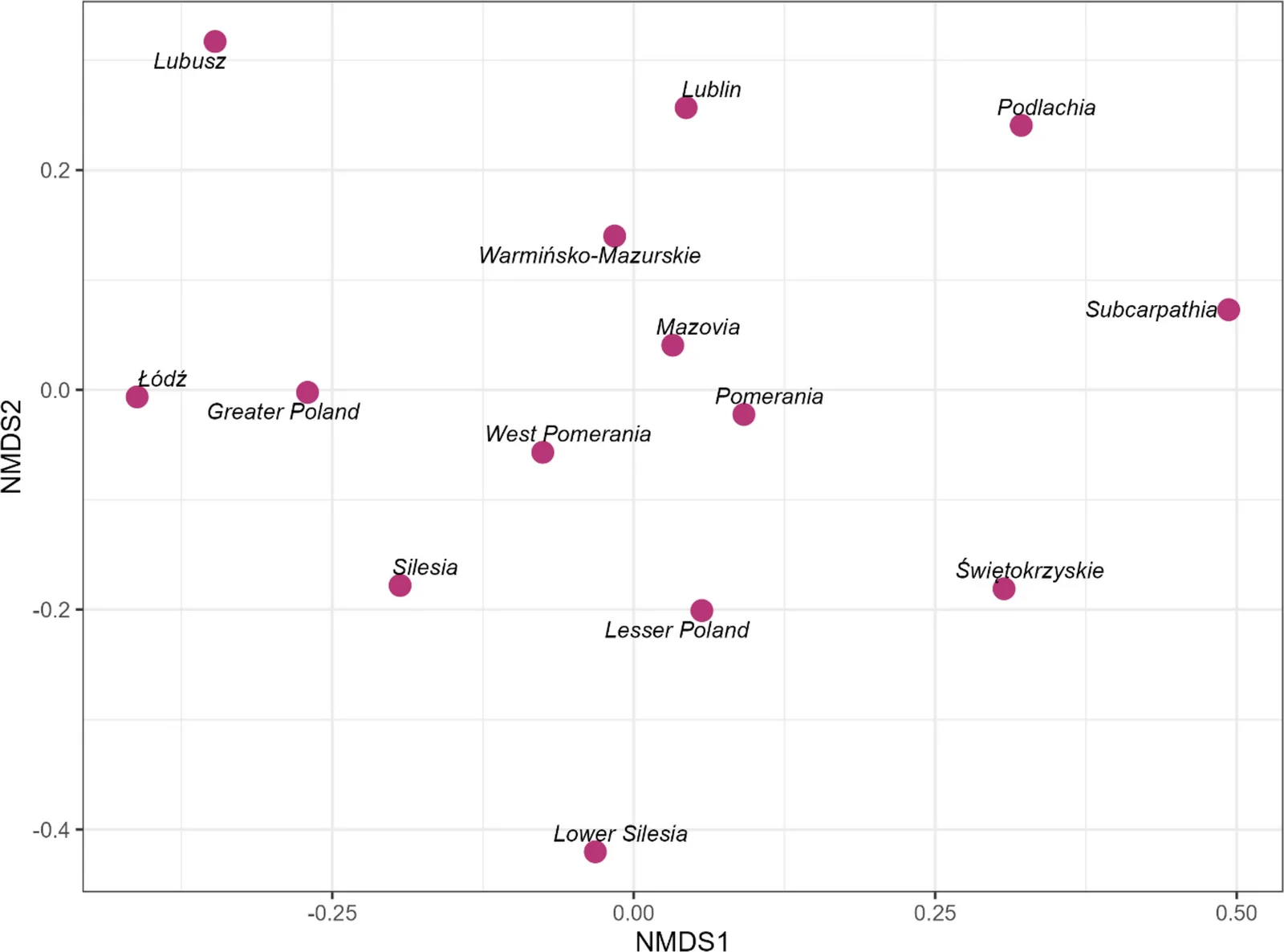
Nonmetric multidimensional scaling (NMDS) of moss species composition across Polish voivodeships (stress = 0.155, including only regions with *n* ≥ 300, nonmetric fit *R*^2^ = 0.976; Supplementary Figure S1), based on a Bray–Curtis dissimilarity matrix

Nonmetric multidimensional scaling (NMDS) analysis revealed variation in moss species composition across the voivodeships (stress = 0.155). Along the NMDS1 axis, the Subcarpathian voivodeship occupied an extreme position, while the Łódź, Lubusz, and Greater Poland voivodeships were located on the opposite side of the ordination space. The Masovian, Lesser Poland, Silesian, and West Pomeranian voivodeships formed a cluster in the central part of the plot (Fig. 7).

Data analyses allowed for the identification of taxa whose occurrence in the dataset was restricted exclusively to a single voivodeship. In the Subcarpathian voivodeship, 10 records of *Hookeria lucens*, 7 observations of *Hylocomiastrum umbratum*, and 3 observations of *Hylocomiastrum pyrenaicum* were recorded. Conversely, the occurrence of *Dicranum majus* (7 records), among others, was documented in the Pomeranian voivodeship.

**Fig. 8:**
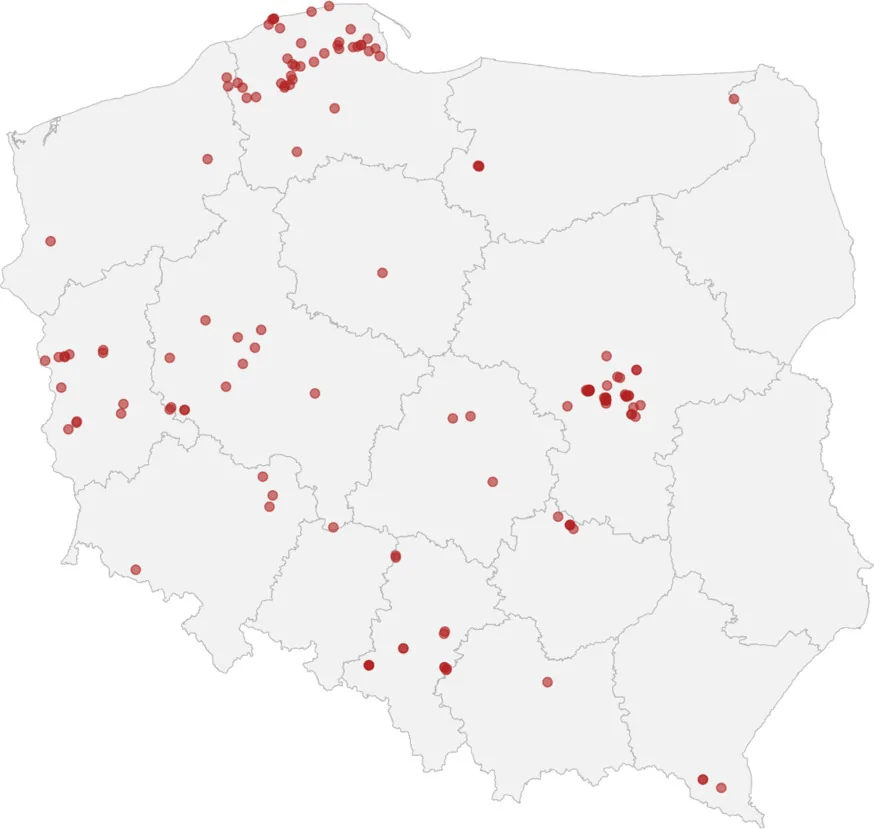
Occurrence of *C*. *introflexus* in Poland based on iNaturalist data (2019–2025)

Regarding the monitoring of alien species, a total of 151 unique records of the invasive moss *Campylopus introflexus* from Poland were identified within the analyzed database. The presence of this species was confirmed in 14 voivodeships (Fig. 8), with the highest number of such records documented in the Masovian voivodeship (48).

### Data validation and limitations of photographic identification

Manual revision of a 9.4% random sample of records (*N* = 1654) indicated visual concordance for easily recognizable taxa, while highlighting the limited ability to assess species requiring microscopic verification. This phenomenon entails a high visual concordance of macroscopic features visible in photographs with the taxonomic diagnosis, coupled with an inability to definitively confirm species that require microscopic analyses. 

Macroscopic concordance between the photographic documentation and the assigned taxon was observed in 70.4% (1165) of cases. However, it should be emphasized that this result primarily reflects the high reliability of documenting species with a characteristic habit. Evident factual errors, involving misidentification, constituted only 0.2% (3) of the sample. The remaining 29.4% (486) was classified as uncertain records, where the photographic documentation inherently precluded the verification of diagnostic features (e.g., *Anomodon*, *Hypnum*, and *Dicranoweisia*).

Interviews with 12 community leaders (defined as the observers with the highest number of contributed records), achieving a 100% response rate, revealed a diverse profile and skill set within this group, which partially explains how they navigate this methodological barrier. Seven respondents declared having relevant formal education (in natural sciences or forestry), and half confirmed the regular use of microscopic verification or a hand lens, which is crucial for identifying more difficult records. While some respondents base their identifications on professional taxonomic keys, others rely primarily on field experience, community verification, and AI algorithms (which, in turn, can exacerbate the aforementioned inaccuracies).

## Discussion

### Dominance structure and perceptual limitations

The high value of the Gini coefficient (*G*_users_ = 0.89) and the composition of the most frequently recorded taxa list suggest the presence of an observational bias in citizen science. Observers show a strong preference for macroscopic species with a characteristic habit that are relatively easy to identify in the field, such as *Pseudoscleropodium purum* or *Pleurozium schreberi* (Cerrejón et al., 2024). The high frequency of records for specific taxa highlights a necessary distinction between species genuinely well suited to photographic identification and those whose representation is driven by observer familiarity or platform tendencies. Widespread and structurally distinct forest species such as *P*. *schreberi* or *Hylocomium splendens* possess robust macroscopic traits that allow for reliable field recognition from photographs. In contrast, high representation in other groups can reflect identification practices rather than true ecological dominance. For instance, the high frequency of *Hypnum cupressiforme* largely stems from its role as a familiar, generalized morphotype to which observers and automated identifiers frequently assign similar-looking pleurocarpous mosses in the absence of microscopic confirmation. An equally important factor is the ease of access to habitats; the high rank of synanthropic species reflects users’ tendency to document taxa in densely populated areas and on anthropogenic substrates (Di Cecco et al., 2021; Geldmann et al., 2016).

In contrast to mosses with large gametophytes, minute species are significantly underrepresented in the iNaturalist database. This is due to the difficulty of photographing them without specialized optical equipment, which most users do not possess. As demonstrated by Arazy and Malkinson (2021), in unstructured citizen science projects, it is equipment limitations that directly determine the ability to detect and report specific groups of organisms. The situation is further exacerbated by the lack of accessible moss identification keys dedicated to amateurs (Cerrejón et al., 2024; McMullin & Allen, 2022). Consequently, the iNaturalist platform effectively documents mainly the so-called “visual fraction” of the flora, while taxa requiring a microscopic analysis of diagnostic features remain the domain of scientific institutions (Cerrejón et al., 2024; McMullin & Allen, 2022) or are misidentified.

Despite these limitations, the temporal analysis (Fig. 2) indicates a clear increase in the number of observations during the analyzed period. While the data in 2019 were incidental, the year 2025 confirms that iNaturalist has become a significant tool for the mass collection of data on moss distribution in Poland by hobbyists. Data from the iNaturalist platform reveal that citizen scientists moss monitoring in Poland is concentrated in two seasons: spring and autumn. The dominance of March as the month with the highest number of records may result from the visibility of mosses during this period. At this time, due to high humidity and the lack of foliage on trees and grasses, mosses are best exposed and easiest to photograph. By becoming the dominant visual element of the ecosystem during the dormancy period of vascular plants, mosses enable data collection during so-called “dead periods,” which have been less frequently explored in traditional floristic studies (Boakes et al., 2016; Cerrejón et al., 2024; Di Cecco et al., 2021; Vanderpoorten & Goffinet, 2009).

### Specificity of the observer community: bryology and the “super-user effect”

Spatial analysis (Fig. 3) confirms the presence of a strong spatial bias (Callaghan et al., 2019); however, its nature in citizen bryology differs from the trends observed in popular branches of citizen science, such as ornithology (Geldmann et al., 2016). In contrast to bird monitoring, which engages thousands of volunteers in Poland who report common species in a relatively even manner, citizen science moss monitoring is a niche activity, largely due to the substantial taxonomic and technical barriers associated with bryophyte identification. This phenomenon is also likely associated with the widespread plant blindness in society (Johnston et al., 2021; Wandersee & Schussler, 1999).

The collected material demonstrates the dominant influence of the so-called “super-user effect” (Boakes et al., 2016). The exceptional position of the Pomeranian Voivodeship (both in terms of the number of records, *n* = 4015, and raw species richness, *S* = 168) does not solely result from the natural values of the region, but is largely a consequence of the intensive activity of a small number of users. On a national scale, the community regularly identifying mosses on the iNaturalist platform numbers merely a few dozen individuals. As a result, distribution maps largely reflect the spatial footprint of a few citizen experts (Isaac & Pocock, 2015), and “blank spots” (e.g., in the Opole Voivodeship) may reflect a lack of local observers rather than an actual poverty of the bryoflora (Geldmann et al., 2016). This phenomenon should be considered an inherent feature of monitoring taxonomically difficult groups (Boakes et al., 2016; Sastre & Lobo, 2009).

The high Spearman’s rank correlation between the number of observations and species richness (rs = 0.86, *p* < 0.001) confirms that the iNaturalist platform, in its current stage of development, primarily documents the field activity of users rather than the actual ranges of individual taxa. As suggested by Isaac and Pocock (2015), without accounting for this relationship, diversity maps de facto become maps of population density or the popularity of tourist routes. Only the application of rarefaction allowed for the separation of the influence of variable observational effort from the true biological signal (Gotelli & Colwell, 2001). Although the Pomeranian Voivodeship maintained its leading position in the standardized ranking, rarefaction drastically reduced the gap between it and the Subcarpathian Voivodeship, revealing the exceptionally high diversity of the latter region per unit of effort invested.

It is worth noting that the high concentration of activity around a few leaders (*G*_users_ = 0.89) is a characteristic feature of citizen sciences with a high entry threshold, to which bryology belongs (Boakes et al., 2016). This stands in stark contrast to the asymmetry in the abundance distribution of the taxa themselves (*G*_taxa_ = 0.76), suggesting that observations are concentrated in a relatively small subset of visually conspicuous species.

In this context, the “super-user effect” can be interpreted not as a systematic error but as a mechanism of informal curation. A few highly active observers act as a quality filter, which, in taxonomically difficult groups, promotes greater reliability of the database (Austen et al., 2016; Johnston et al., 2018).

While the high unstandardized species count for the Pomeranian Voivodeship results primarily from the enormous sampling effort, standardized richness estimations confirm its high regional diversity. This is further supported by the actual presence of regionally rare taxa, such as *Dicranum majus* (Wierzgoń & Skowron, 2024). On the other hand, the Subcarpathian Voivodeship achieved an almost identical diversity index (*H*′ = 4.21) with three times less sampling effort (*n* = 1112) than the central Masovian Voivodeship (*n* = 3224). This confirms that in regions of high naturalness, such as the Carpathian Forest or the Bieszczady Mountains, citizen science data can effectively detect rare components of the flora (Tulloch et al., 2013), provided that at least one active observer contributes records from the region.

Particularly interesting is the contrast between the Pomeranian and West Pomeranian voivodeships. Despite the similarity in physical–geographical conditions (Solon et al., 2018), the distinct difference in the number of recorded taxa suggests an underrepresentation of the western region, resulting from the lack of a local community leader. This indicates that iNaturalist data currently reflect the intensity of sampling effort (effort bias) rather than definitive biogeographical boundaries (Geldmann et al., 2016).

These conclusions are reflected in the results of the ordination analysis (Fig. 7). Voivodeships such as Łódź or Lubusz group far away from, e.g., the Subcarpathian Voivodeship, which is not evidence of their natural uniqueness, but the effect of insufficient data saturation, causing individual observations to strongly project onto the region’s position in the analyses (Gotelli & Colwell, 2001). At low data saturation, the representation of the flora depends on a few random observations, causing artifacts that affect its position in multidimensional space. The position of the Subcarpathian region should be interpreted quite differently—its ordination distinctiveness results from the documentation of indicator and lower montane zone species, such as *Hookeria lucens* (Stebel et al., 2004), or species from the genus *Hylocomiastrum* (Stebel & Żarnowiec, 2014).

### Potential in monitoring rare and indicator species

The study demonstrated the significant potential of the iNaturalist platform in documenting taxa with a characteristic, a unique habit, which allows for partially overcoming the taxonomic barriers typical for this plant group (Cerrejón et al., 2024). Recording 10 localities of *Hookeria lucens* in the Subcarpathian Voivodeship—a species considered an indicator for forests with a high degree of naturalness and a preserved primeval structure (Stebel et al., 2004)—confirms that users of platforms such as iNaturalist can effectively support the monitoring of rare mosses (Wilson et al., 2020). These results suggest that citizen science data can provide a valuable supplement to official databases on the distribution of protected and threatened species, provided, however, that mass observations—which remain uncertain due to the imperfections of artificial intelligence algorithms regarding cryptogams—are subjected to additional expert verification (Munzi et al., 2023).

The platform offers equally significant possibilities in the field of monitoring biological invasions. Due to the great ease of macroscopic identification, mosses such as *Campylopus introflexus* become ideal subjects for crowdsourcing research (Cerrejón et al., 2024; Hiller & Haelewaters, 2019). iNaturalist allows for tracking the directions and rate of expansion of invasive species in near real-time, which often precedes traditional publication cycles and reporting based on classical floristic inventories (Genovesi et al., 2015). The results of this study constitute a direct continuation and confirmation of the trends described by Żarnowiec et al. (2018). Summarizing 30 years of invasion (1986–2016), these authors demonstrated the presence of *C*. *introflexus* at 248 localities, documenting its expansion from the west to the east of the country. The fact that between 2019 and 2025, the iNaturalist platform enabled the documentation of as many as 151 verified unique records across 14 voivodeships highlights the widespread occurrence of this invasive species and demonstrates the potential of citizen science in detecting invasive or alien species. Particularly telling is the high number of records in the Masovian Voivodeship (48 records), which confirms the accuracy of the predictions by Żarnowiec et al. (2018) regarding the colonization of the lowland parts of central and eastern Poland. This model fits into modern biodiversity management strategies, in which mass citizen science data serve as an Early Warning System (Encarnação et al., 2021; Groom et al., 2015). However, the effectiveness of this approach depends on the continuous moderation of the database by experts, which allows for the elimination of misidentifications of morphologically similar species (Austen et al., 2016; Munzi et al., 2023).

### Methodological limitations

It is worth emphasizing that this analysis is based on photographic documentation, which defines the specific interpretive scope of citizen science data in bryology. Mosses, unlike most vascular plants, are characterized by a unique diagnostic structure in which a significant portion of differentiating features is microscopic (Vanderpoorten & Goffinet, 2009). The exclusive use of digital records, without direct access to herbarium material, shifts the focus of the analysis from microscopic features toward habit-related traits (McMullin & Allen, 2022).

This phenomenon is associated with the occurrence of so-called “cryptic diversity” (Cerrejón et al., 2024), where phenotypically similar taxa can be difficult to distinguish at the level of a photograph alone, which is noticeable, for instance, within the genus *Bryum* (Holyoak, 2004). An extremely telling illustration of this problem in the Polish iNaturalist database is the case of the genus *Hypnum*. Although 14 species of *Hypnum* are reported from Poland (Ochyra et al., 2003), only two species (*H*. *cupressiforme* and *H*. *jutlandicum*) were retained in the analytical dataset. *Hypnum andoi* was represented by a single verified observation and was excluded during singleton filtering. This illustrates both the rarity of some taxa in citizen science datasets and the limited ability of photographic observations to capture the full diversity of taxonomically challenging genera. Thus, the analytical dataset captured only 14.3% of the known national diversity of the genus, while the original Research Grade dataset captured 21.4%, suggesting a substantial reduction of taxonomic complexity almost to a single visually recognizable morphotype. Although singleton taxa were excluded from the analytical dataset, all such records were manually reviewed prior to analyses. As a result, a small number of genuinely rare species may also have been omitted, including *Splachnum ampullaceum*, which was documented by only a single verified record (Supplementary Table S2).

The lack of microscopic verification may contribute to a tendency to assign many morphologically similar observations to the most familiar and widespread taxon, *H*. *cupressiforme*. This is a specific limiting factor because the platform excels at recording the presence of general morphotypes, but without a microscope, it loses the actual species diversity within them. This phenomenon clearly demonstrates that community consensus in the case of mosses often merely signifies agreement on macroscopic appearance rather than actual taxonomic identity. Consequently, for many moss taxa, the “Research Grade” status on iNaturalist should be interpreted primarily as confirmation of agreement regarding visible macroscopic features rather than definitive species-level verification. It constitutes a taxonomic approximation, allowing for the documentation of those elements of the flora that can be identified based on macroscopic traits, even if it does not fully replace classical microscopic analyses. 

A key challenge in utilizing iNaturalist data is an identification error resulting from the limitations of photography itself. However, the manual verification of almost a 10% random sample of records (*n* = 1654), conducted in this study, revealed a certain paradox. On the one hand, it demonstrated high apparent reliability of the database (macroscopically correct identification in 70.4% of cases)—which proves that the Polish community handles the “visual fraction” well (e.g., *Polytrichum piliferum*). On the other hand, during verification, we rely on the assumption that what is visible in the photograph is sufficient. For a significant portion of mosses, such as representatives of the genus *Plagiothecium* (Wolski, 2025) or *Hypnum* (Smith, 2004), this is a false assumption, because the key diagnostic features lie at the level of microscopic cell structure. As a result, even the correct verification of such a photograph by an expert only confirms the visual consistency of the specimen with a given taxon, and does not provide ultimate biological certainty regarding the species. Despite this methodological constraint, the validation procedure proves that with the application of appropriate filters (Research Grade) and awareness of the limits described above, citizen science can potentially provide useful data supplementing classical floristic inventories (Kosmala et al., 2016).

Despite this methodological specificity, mass amateur data are becoming an important complement to modern biodiversity monitoring in an era of dynamic environmental changes. According to European reports, invasive alien species (IAS) currently constitute one of the main threats to native biodiversity (European Environment Agency, 2020). In this context, the iNaturalist platform, by providing near real-time information on relatively easily recognizable taxa (such as *C*. *introflexus*), serves as an early warning system. A hybrid model, combining the mass scale of citizen science observations with professional expert verification, delineates a promising direction for the development of bryology today, allowing for more effective tracking of nature’s response to modern threats (Dickinson et al., 2010). Furthermore, clusters of records of hygrophilous or calciphilous species can serve as a specific indicator of priority areas for specialists, pointing to locations of potentially rare habitats, such as previously unknown mid-forest peat bogs or springs. As a result, even observations of more common species, but with a narrow ecological niche, become a tool for identifying valuable ecosystems that might otherwise remain unnoticed in traditional inventories.

While our study focused primarily on structured metadata, the unstructured 'Notes' section associated with iNaturalist observations represents a highly valuable, albeit currently underutilized, resource. For bryophytes, explicit documentation provided by observers regarding the substrate (e.g., bark, soil, rock, and decaying wood) and its broader ecological context would significantly enhance the value of citizen science data for interpreting species occurrences and habitat associations. To further maximize this research value and overcome the “microscope barrier,” future efforts should prioritize independent validation of representative record subsets by professional bryologists. Based on our findings, we propose three practical recommendations for the iNaturalist community: (1) photographing key diagnostic structures (e.g., capsules, leaf margins, and growth forms) from multiple angles; (2) consistently utilizing the observation notes to document the substrate; and (3) clearly indicating in the comments when an identification was supported by microscopic examination (ideally including microphotographs as evidence) or specialized literature.

## Summary and conclusions

Analysis of iNaturalist data from 2019 to 2025 demonstrates that citizen science can effectively support moss monitoring in Poland. Observer activity increased over time, reaching distinct peaks in spring and autumn. However, the data structure was strongly dependent on the activity of a small group of users, highlighting the need for methods standardizing observational effort. The platform proved particularly effective in documenting easily recognizable species, including the invasive *C*. *introflexus*. At the same time, limitations of photographic identification mean that the data mainly represent the “visual fraction” of the bryoflora. Despite these limitations, iNaturalist may serve as an early-warning system and help identify priority areas for further bryological research. Ultimately, while iNaturalist data significantly support the large-scale mapping of selected, conspicuous mosses, they must be treated as a valuable complement to, rather than a replacement for, traditional professional bryological surveys—especially within critical taxonomic groups that inherently demand microscopic validation.

## Supplementary Information

Below is the link to the electronic supplementary material.

ESM 1: Supplementary Figure S1 (PNG 39.7 KB)

ESM 2: Supplementary Code S1 (R 14.6 KB)

ESM 3: Supplementary Material S1 (CSV 17.5 MB)

ESM 4: Supplementary Table S1 (CSV 575 bytes)

ESM 5: Supplementary Table S2 (CSV 5.14 KB)

## Data Availability

The raw iNaturalist dataset supporting the conclusions of this article (Supplementary Material S1) and the custom R script used for all analyses and figure generation (Supplementary Code S1) are available online. Additional supporting materials include: • Supplementary Table S1: Summary of moss diversity metrics and observer bias across Polish voivodeships (2019–2025). • Supplementary Table S2: A list of the 45 excluded singleton observations, including geographic coordinates and hyperlinks to the original photographic documentation. • Supplementary Figure S1: Shepard diagnostic plot assessing the goodness-of-fit for the non-metric multidimensional scaling (NMDS) ordination.
